# Somatic RIT1 delins in arteriovenous malformations hyperactivate RAS-MAPK signaling amenable to MEK inhibition

**DOI:** 10.1007/s10456-024-09934-8

**Published:** 2024-07-05

**Authors:** Friedrich G. Kapp, Farhad Bazgir, Nagi Mahammadzade, Mehrnaz Mehrabipour, Erik Vassella, Sarah M. Bernhard, Yvonne Döring, Annegret Holm, Axel Karow, Caroline Seebauer, Natascha Platz Batista da Silva, Walter A. Wohlgemuth, Aviv Oppenheimer, Pia Kröning, Charlotte M. Niemeyer, Denny Schanze, Martin Zenker, Whitney Eng, Mohammad R. Ahmadian, Iris Baumgartner, Jochen Rössler

**Affiliations:** 1 Division of Pediatric Hematology and Oncology, Department of Pediatrics and Adolescent Medicine, Medical Center-University of Freiburg, Faculty of Medicine, University of Freiburg, VASCERN VASCA European Reference Centre, 79106 Freiburg, Germany; 2 Institute of Biochemistry and Molecular Biology II, Medical Faculty and University Hospital, Heinrich-Heine University, Düsseldorf, Germany; 3 Institute of Pathology and Tissue Medicine, University of Bern, Bern, Switzerland; 4 Division of Angiology, Swiss Cardiovascular Center, Inselspital, Bern University Hospital, Bern, Switzerland; 5 Department for BioMedical Research (DBMR), University of Bern, Bern, Switzerland; 6 Institute for Cardiovascular Prevention (IPEK), Ludwig-Maximilians University Munich, Pettenkoferstr 9, 80336 Munich, Germany; 7 Vascular Biology Program, Department of Surgery, Boston Children’s Hospital, Harvard Medical School, Boston, MA USA; 8 Department of Pediatrics and Adolescent Medicine, Friedrich-Alexander-Universität Erlangen-Nürnberg (FAU), 91054 Erlangen, Germany; 9 Department of Otorhinolaryngology, Regensburg University Medical Center, Franz-Josef-Strauß-Allee 11, 93053 Regensburg, Germany; 10 Department of Radiology, Regensburg University Medical Center, Franz-Josef-Strauß-Allee 11, 93053 Regensburg, Germany; 11 University Clinic and Policlinic of Radiology at the Martin-Luther-Universität Halle-Wittenberg, Halle, Germany; 12 Department of General Pediatrics, Adolescent Medicine and Neonatology, Medical Center-University of Freiburg, Faculty of Medicine, University of Freiburg, 79106 Freiburg, Germany; 13 Institute of Human Genetics, University Hospital Magdeburg, 39120 Magdeburg, Germany; 14 Division of Hematology/Oncology, Boston Children’s Hospital and Harvard Medical School, Boston, MA USA; 15 Department of Vascular Medicine, National Reference Center of Rare Lymphatic and Vascular Diseases, UA11 INSERM – UM IDESP, Campus Santé, Montpellier Cedex 5, France; 16 Division of Paediatric Hematology and Oncology, Department of Paediatrics, Inselspital, Bern University Hospital, University of Bern, Bern, Switzerland

**Keywords:** Vascular anomalies, Vascular malformation, Arteriovenous malformation, RIT1, RAS-MAPK pathway, Trametinib

## Abstract

Arteriovenous malformations (AVM) are benign vascular anomalies prone to pain, bleeding, and progressive growth. AVM are mainly caused by mosaic pathogenic variants of the RAS-MAPK pathway. However, a causative variant is not identified in all patients. Using ultra-deep sequencing, we identified novel somatic *RIT1* delins variants in lesional tissue of three AVM patients. *RIT1* encodes a RAS-like protein that can modulate RAS-MAPK signaling. We expressed *RIT1* variants in HEK293T cells, which led to a strong increase in ERK1/2 phosphorylation. Endothelial-specific mosaic overexpression of *RIT1* delins in zebrafish embryos induced AVM formation, highlighting their functional importance in vascular development. Both ERK1/2 hyperactivation in vitro and AVM formation in vivo could be suppressed by pharmacological MEK inhibition. Treatment with the MEK inhibitor trametinib led to a significant decrease in bleeding episodes and AVM size in one patient. Our findings implicate *RIT1* in AVM formation and provide a rationale for clinical trials with targeted treatments.

**Supplementary Information:**

The online version contains supplementary material available at 10.1007/s10456-024-09934-8.

## Introduction

Vascular anomalies are classified according to the Classification of the International Society for the Study of Vascular Anomalies (ISSVA) and are subdivided into vascular tumors and vascular malformations [1, 2]. While vascular tumors show increased cell proliferation, vascular malformations are thought to represent mainly non-proliferative lesions that originate from errors in vascular development. Most vascular malformations are caused by a somatic mosaic mutation in the affected tissue. Activation of the PI3K-AKT-mTOR pathway are thought to predominate in slow-flow malformations such as venous and lymphatic malformations [3–5], whereas variants activating the RAS-MAPK pathway are typically associated with fast-flow malformations such as AVM [6–9].

Extracranial AVM can occur anywhere in the body, most often in the soft tissue of extremities as well as the head and neck [10, 11]. AVM may become symptomatic with swelling, pain, pulsations, and bleeding. AVM located in the face may lead to major disfigurement and life-threatening complications [12]. Almost all AVM progress over time [13, 14], which may lead to tissue necrosis, bleeding complications, and hyperdynamic heart failure. Treatment is mainly interventional (embolization) and/or surgical resection; however, invasive therapies can activate the lesion and often lead to relapse [14]. However, the exact pathomechanism of AVM development and progression are poorly understood. Taken together, AVM belongs to the most aggressive vascular anomalies and are often difficult to treat, highlighting the need for novel treatment strategies.

In this project, we identified novel mosaic delins variants in *RIT1* that were found in the lesional tissue of three patients with extracranial AVM. *RIT1* acts as modulator of the RAS-MAPK pathway and has so far not been implicated in the development of AVM. We characterized these variants by assessing their effect on ERK phosphorylation in vitro, on vascular development in vivo in a zebrafish model, and the response to MEK inhibition. We further present data on the off-label use of trametinib in one patient.

## Results

### Novel *RIT1* delins variants identified in AVM tissue from three patients

To identify underlying genotypic changes, a total of 691 samples of patients with vascular anomalies underwent next generation sequencing at three different centers for vascular anomalies. Out of these 691 samples, approximately 100 were from patients with an AVM. Three patients with an extracranial AVM were found to harbor a *RIT1* mutation.

**Fig. 1:**
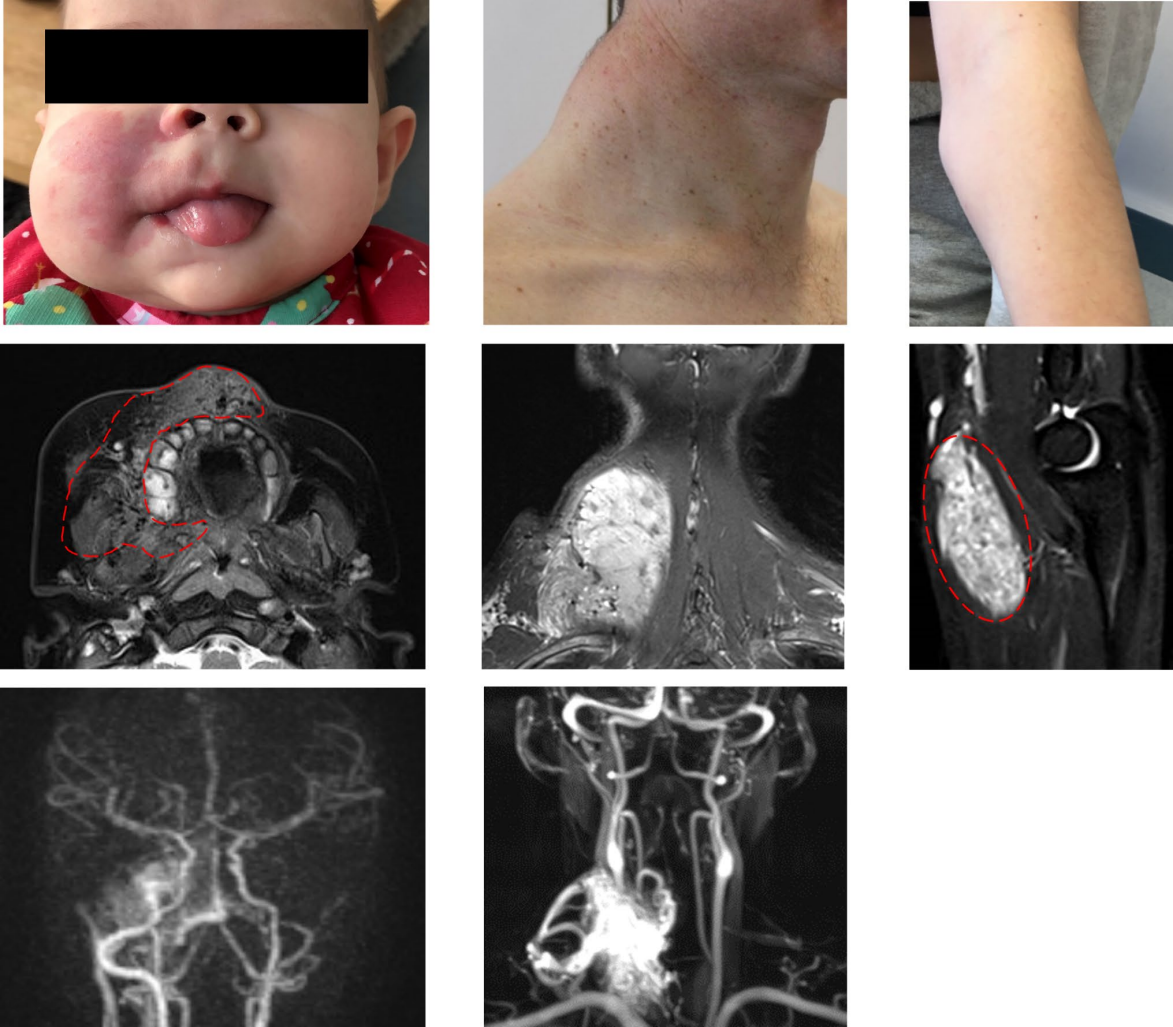
Three patients with somatic *RIT1* variants identified in AVM tissue. **a** Patient P1 displays a capillary malformation and swelling of the right side of the face. **b** MRI of P1 at the age of 4 months, the image of a transversal T2 TSE sequence, in which an AVM could be detected; the extent of the lesion is labelled with red dashed line indicating soft tissue edema and flow-voids. **c** The MR angiography shows increased perfusion on the right side of the face (left side within the panel). **d** Patient P2 shows a prominent mass of the left cervical/nuchal area. **e** MR imaging (coronal T2 sequence with fat saturation), which shows the hyperintense isolated intramuscular lesion, flow-voids seen within the lesion representing high AVM flow, and disfiguring overgrowth. **f** MR angiography, which shows the large AVM connected to the subclavian and the thyrocervical trunk with multi-chambered central nidus. **g** Clinical aspect of the Patient P3 with swelling on the left forearm, close to the medial side of the elbow. **h** T2W sagittal images demonstrating a well-defined fusiform shaped hyperintense lesion involving the flexor muscle (pronator teres) of the forearm. Flow voids (red dashed line) are seen within the lesion representing arterial blood vessels. *MRI* Magnetic Resonance Imaging

Patient 1 (P1) was a 3-year-old girl with an AVM of the right face. A capillary anomaly and swelling of the right cheek were noticed at birth (Fig. 1A). A diagnosis of an infantile hemangioma was initially made at an external hospital and propranolol was initiated at one month of age. The lesion did not respond to this therapy and a first episode of epistaxis occurred at 6 months of age, eventually leading to the diagnosis of a facial AVM on magnetic resonance angiography (MRA) (Fig. 1B, C). Following this bleeding event, a first catheter embolization with Onyx was performed. Two additional embolizations followed until the age of 22 months, the last intervention of which was combined with bleomycin electro-sclerotherapy [15]. The lesion did not respond to either therapy and the AVM progressed with intermittent life-threatening bleeding episodes (Online Resource 1). Due to progressive symptoms, the patient was then treated with extensive Onyx embolization of the AVM, and a biopsy was obtained for genetic analysis. These interventions, including the removal of a molar that was rooted within the AVM, alleviated the symptoms only slightly. Due to the nature and course of the disease, a hemimaxillectomy was considered. However, infiltration of the AVM into the orbit made a cure by this very invasive approach seem unlikely. We thus initiated off-label treatment with thalidomide (25 mg per day) when the patient was 2 years of age. Under this treatment—and after the last extensive embolization—the severity of bleeding episodes decreased over the next months before deteriorating again. Genetic testing of the biopsy of affected tissue identified a *RIT1* delins variant (c.246_248delinsCCCTCT p.T83delinsPL (referred to as *RIT1*^*P1*^, hereafter)), with a variant allele frequency (VAF) of 3.3%.

Patient 2 (P2) was a 42-year-old man with a first episode of neck pain at the age of 35 years. A continuously growing and pulsating vascular lesion was detected (Fig. 1D). MRA showed an isolated intramuscular AVM connected to the subclavian and the thyrocervical trunk on the right side with disfiguring diffuse muscle involvement including the splenius capitis muscle (Fig. 1E, F). Therapy with sirolimus was initiated but had to be discontinued due to suppurative osteomyelitis of the jaw within 3 weeks. At the age of 39 years, the patient received three direct intraarterial ethanol embolizations at monthly intervals without success. Although the initially dominating nidus of the AVM was completely shut down, there was a massive proliferation of microfistular AV shunts and an increase in tissue volume as a result. One year after embolization, debulking surgery was performed after the situation had stabilized. Histopathology showed typical findings of a diffuse intramuscular microfistular AVM. Ten months later, progression of the AVM was noted again and a combined approach with Onyx embolization and gross total resection was performed. Since then, the patient has been without complaints with stable disease and minimal radiological residuum. A *RIT1* delins variant was identified in the resected tissue (c.242_248delinsTCCCTCT p.E81_T83delinsVPL (referred to as *RIT1*^*P2*^, hereafter) with a VAF of 6.0%.

P3 was a 17-year-old girl, who presented with a persistent prominence in the left forearm that was first noted one year before (Fig. 1G). At the time of the initial presentation, there was no associated pain, no functional deficit, no overlying skin changes, and only minimal swelling. An initial ultrasound was notable for a 5.4 cm × 1.1 cm × 4.7 cm intramuscular mass in the left forearm with diffuse internal vascularity seen on Doppler examination. An MRI of the lesion was notable for a solid enhancing mass in the left pronator teres muscle with imaging findings consistent with a solid neoplasm (Fig. 1H). She underwent an IR-guided biopsy of the lesion. Histopathology was consistent with an intramuscular fast-flow vascular anomaly. She was followed for the next two years and had progressive growth of the lesion associated with pain. Given the worsening of her symptoms, she underwent resection of the lesion. There were no complications and she has had minimal pain since. In the resected tissue, a *RIT1* delins variant was identified (c.229delinsTTGGATACAA p.A77delinsLDTT (referred to as *RIT1*^*P3*^, hereafter) with a VAF of 13%.

### RIT1 delins-induced ERK hyperphosphorylation can be reversed by pharmacological inhibition of MEK but not SHP2

**Fig. 2:**
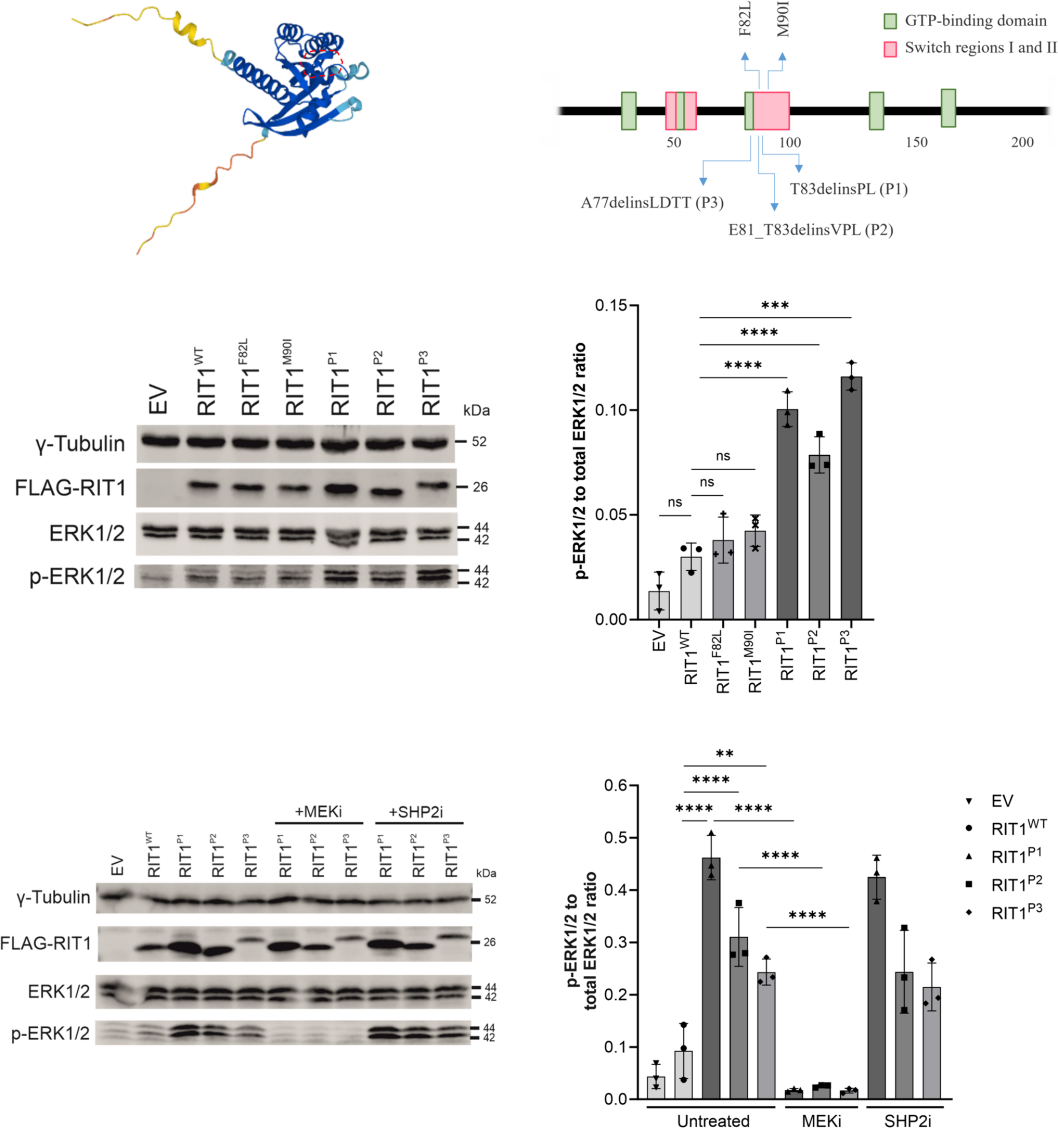
ERK phosphorylation after expression of *RIT1* variants in vitro in HEK293T cells. **a** Protein structure of RIT1 predicted by AlphaFold, accessed through ensemble.org. The area labelled by the dashed red line indicates the switch 2 domain. **b** Schematic drawing of *RIT1* functional domains of human RIT1 protein (green boxes = GTP-binding regions; red boxes = switch domain 1 and 2; blue arrows (upward) = two mutations typically found in Noonan syndrome; blue arrows (downward) = mutations identified in P1-P3. **c** Western blot after expression of *RIT1* variants to assess RAS-MAPK pathway activation. Gamma tubulin served as loading control, FLAG-RIT1 confirms the expression of the construct, total ERK levels serve as a control to exclude the differential expression of ERK, and p-ERK measures the level of phosphorylate of ERK as a marker of RAS pathway activation. **d** Quantification of the ERK phosphorylation was measured in a total of three western blots for each variant (n = 3). One-way ANOVA. P value ***< 0.001; ****< 0.0001, ns = not significant. Data are presented as mean ± SD. EV = empty vector. **e** Western blot after expression of *RIT1* variants and with or without treatment using a MEK inhibitor or SHP2 inhibitor. The same parameters were assessed as in panel d. **f** Quantification of the ERK phosphorylation was measured in a total of three western blots for each variant (n = 3). One-way ANOVA. P value **< 0.01; ****< 0.0001, ns = not significant. Data are presented as mean ± SD

All three delins variants are located close to the switch 2 domain of the RIT1 protein, a region that also harbors germline missense variants commonly associated with Noonan syndrome (Fig. 2A, B). To investigate the impact of these novel mutations on activation of the RAS pathway, we assessed ERK phosphorylation by Western blotting after the expression of *RIT1* in the HEK293T cells. We expressed *RIT1*^*P1*^*, RIT1*^*P2*^*, RIT1*^*P3*^*, RIT1* wildtype (*RIT1*^*wt*^), and two recurrent *RIT1* mutations found in Noonan syndrome (p.F82L and p.M90I). All three novel *RIT1* delins led to a significant increase in ERK phosphorylation, while overexpression of the two Noonan syndrome-associated *RIT1* mutations only induced a modest ERK hyperphosphorylation (Fig. 2C, D). Additionally, we examined the effects of the *RIT1* variants on PI3K/AKT signaling pathway activation by assessing the ratios of p-AKT (Thr308) as a substrate of PDK1 and p-AKT (Ser473) as the target of mTORC2 to the total levels of AKT. Interestingly, higher levels of phosphorylation at AKT-Threonine-308 were observed in the AVM associated delins, while the phosphorylation at Serine-473 remained unaffected (Online Resource 2).

Since RAS proteins act downstream of SHP2 and upstream of MEK and ERK in the RAS-MAPK signaling pathway, we hypothesized that RIT1-induced ERK hyperphosphorylation exhibits a differential response to treatment with SHP2 and MEK inhibition (Online Resource 3). Indeed, treatment of HEK293T cells with the SHP2 inhibitor SHP099 showed no effects on ERK phosphorylation. In contrast, MEK inhibition with PD0325901 reversed ERK phosphorylation close to baseline levels (Fig. 2E, F).

### *RIT1* delins variants lead to the formation of AVM-like lesions in zebrafish embryos

**Fig. 3:**
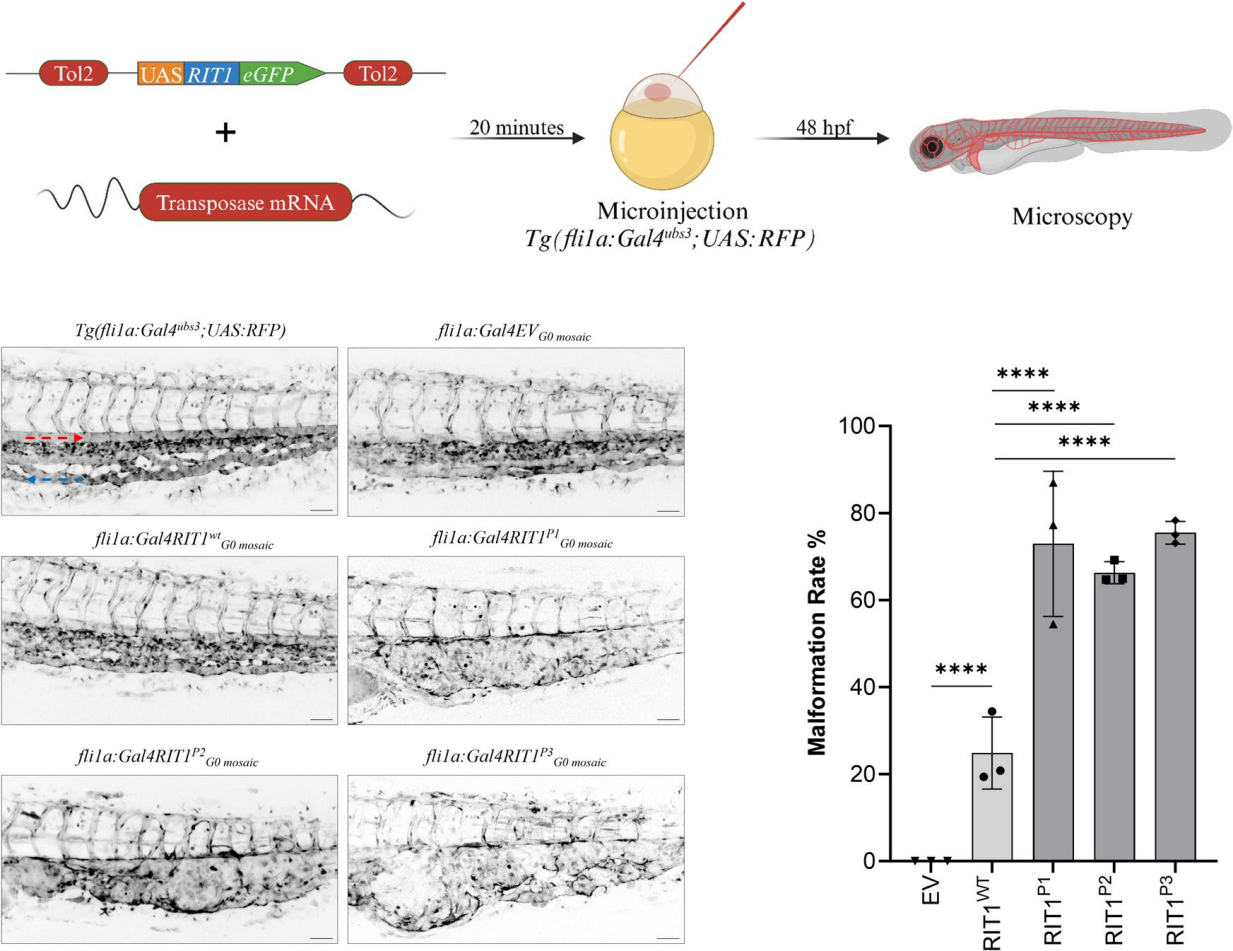
Endothelial-specific mosaic expression of *RIT1* variants leads to the formation of AVM in zebrafish embryos. **a** Experimental layout: The plasmid containing human wildtype *RIT1* or *RIT1* variants, under the control of a UAS element and linked to GFP with a P2A sequence is mixed with transposase mRNA and injected into the one-cell stage of *Tg(fli1a:Gal4; UAS:RFP)* embryos. Thereafter, embryos are examined at 48 hpf. **b** Vascular network in the tail of an uninjected *Tg(fli1a:Gal4; UAS:RFP)*, EV and *RIT1* variants injected embryos. Arrows represent the direction of arterial and venous blood flow (red and blue arrow, respectively). Note the malformed vasculature with a fusion of the dorsal aorta and the caudal vein as well as dilation of the vessel. Scale bar 50 µm. **c** Quantification of the vascular anatomy at 48 hpf following the injection of plasmids containing the indicated *RIT1* variants, with and without treatment. n = 3. Number of total examined embryos: EV = 66, *RIT1*^*WT*^ = 92, *RIT1*^*P1*^ = 67, *RIT1*^*P2*^ = 97, *RIT1*^*P3*^ = 61. Fisher’s exact test, two-tailed. P value ****< 0.0001. Data are presented as mean ± SD. *EV* empty vector

**Fig. 4:**
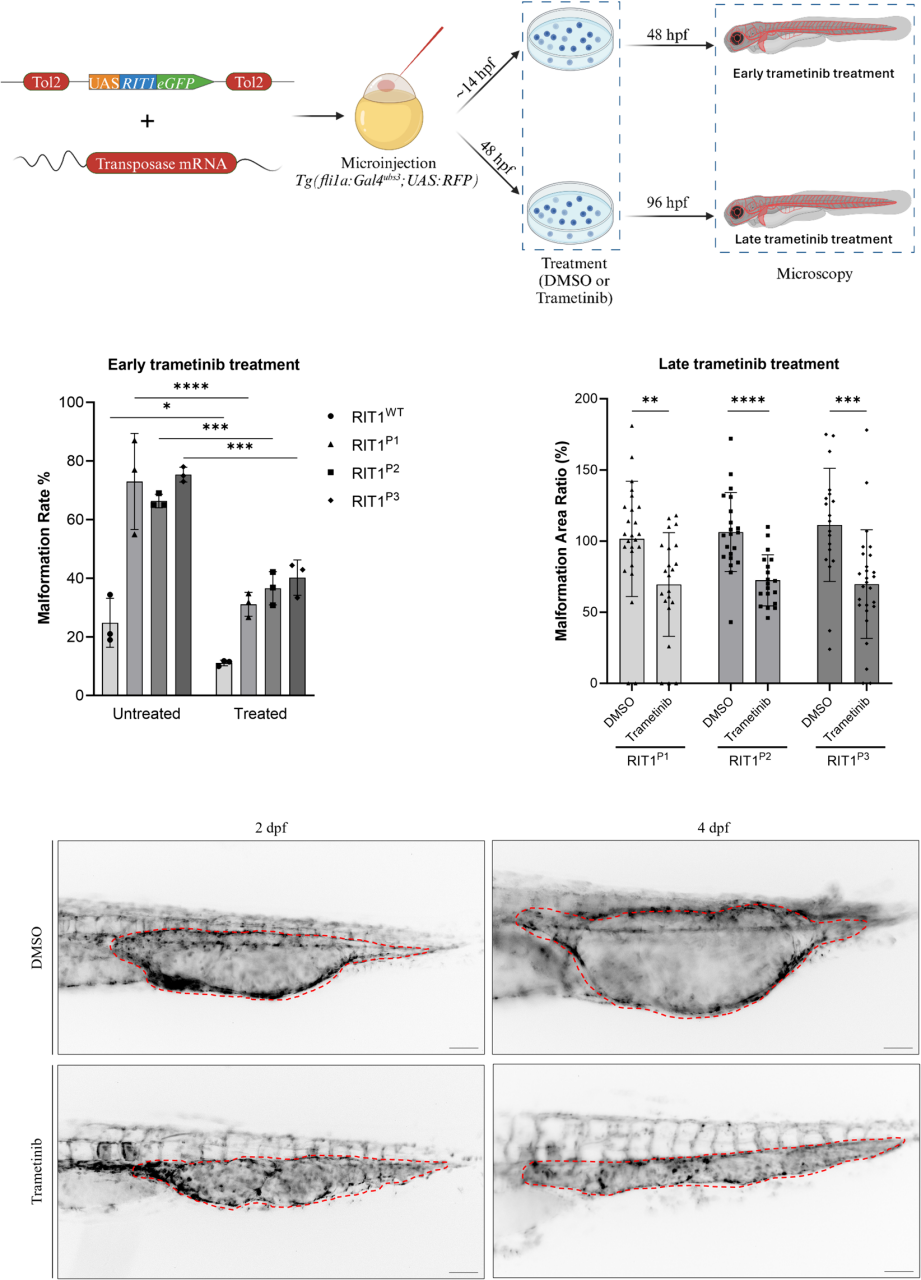
*RIT1* variants injected zebrafish embryos respond to early and late treatment with MEK inhibitor trametinib. **a** Experimental plan to assess the effect of trametinib on the *RIT1* injected zebrafish embryos after early and late treatments. Embryos are treated with trametinib either from 14hpf or 48 hpf on followed by an examination at 48 hpf (early treatment) or 96 hpf (late treatment) respectively. Presence of vascular malformation is calculated for early treatment embryos. Malformation area is calculated before and after trametinib treatment for each embryo in the late treatment group.** b** Quantification of the vascular anatomy at 48 hpf following the injection of plasmids containing the indicated *RIT1* variants and treatment at 14 hpf. Experiments were performed with three biological replicates. Number of total examined embryos post-treatment: *RIT1*^*WT*^ = 73, *RIT1*^*P1*^ = 60, *RIT1*^*P2*^ = 51, *RIT1*^*P3*^ = 38. Fisher’s exact test, two-tailed. P value *< 0.05, ***< 0.001; ****< 0.0001. Data are presented as mean ± SD.** c** Quantification of the relative change in the size of the AVM-like lesions after 2 days of treatment. Experiments were performed with three biological replicates. Number of total examined embryos: *RIT1*^*P1*^ = 48, *RIT1*^*P2*^ = 40, *RIT1*^*P3*^ = 46. Unpaired t-test, two-tailed. P value **< 0.01; ***< 0.001; ****< 0.0001. Data are presented as mean ± SD.** d** Example of image analysis to measure the relative change of lesion size during trametinib treatment from 2 to 4 dpf. Scale bar 50 µm

Having shown that the *RIT1* delins identified in AVM patients induced strong activation of the RAS-MAPK pathway, we next assessed whether their expression can lead to aberrant vascular development in the tail vasculature of zebrafish embryos. The zebrafish is an established model for the study of vascular development [16] that has also been applied to translational research [8, 17, 18]. To this end, we used plasmids, which contain the transcriptional upstream activating sequence (UAS) that controls the expression of wildtype or variant *RIT1* linked to GFP via the self-cleaving peptide P2A. Activation of the UAS element and thus expression of *RIT1* is dependent on the presence of the transcription factor Gal4. These plasmids were injected in the one-cell stage of *Tg(fli1a:Gal4; UAS:RFP)* embryos. While the plasmid integrated randomly into the DNA of cells of the zebrafish embryo, expression of RIT1-P2A-GFP was limited to endothelial cells that expressed Gal4 under the control of the endothelial fli1a promoter (Fig. 3A) [18]. Using this approach, we observed AVM-like lesions in the zebrafish embryo tail at 2 dpf. These lesions were characterized by aberrant connections between the dorsal aorta and the caudal vein. A common and severe phenotype exhibited a fusion of these arterial and venous vessels (Fig. 3B, Online Resource 4). In many embryos, the aorta and part of the caudal vein plexus directly downstream of the shunt at the proximal end of the AVM-like lesions were also fused, but a shunt could also be focal only (Online Resource 5 and 6). This fusion is in line with a recent study on *rasa1* mutant zebrafish with a similar phenotype of a distal fusion [19]. A significantly higher rate of AVM-like lesions at 48 h post fertilization (hpf) was observed in embryos expressing *RIT1*^*P1−P3*^ delins compared to *RIT1*^*wt*^ (66–75% vs 24%, Fig. 3C). Next, we treated injected embryos with 100 nM trametinib during early development. This early treatment significantly reduced the formation of AVM-like lesions (31–39%, Fig. 4A, B), thereby supporting the assumption that AVM formation is critically dependent on hyperactivation of the RAS-MAPK pathway. To mimic a targeted treatment more closely in patients, we next treated zebrafish embryos with established AVM-like lesions after injection of *RIT1* delins with 100 nM trametinib from 48 hpf onwards and compared growth of the lesions in treated and untreated embryos over the following two days. DMSO-treated embryos in the control group showed a relative increase in the size of the lesion to 117.5%, while the size of the lesions in trametinib-treated embryos decreased to 83.4%; the difference between treated and untreated embryos was significant (Fig. 4C, D, Online Resource 7).

### Trametinib induced reduction in AVM size and bleeding frequency in P1

**Fig. 5:**
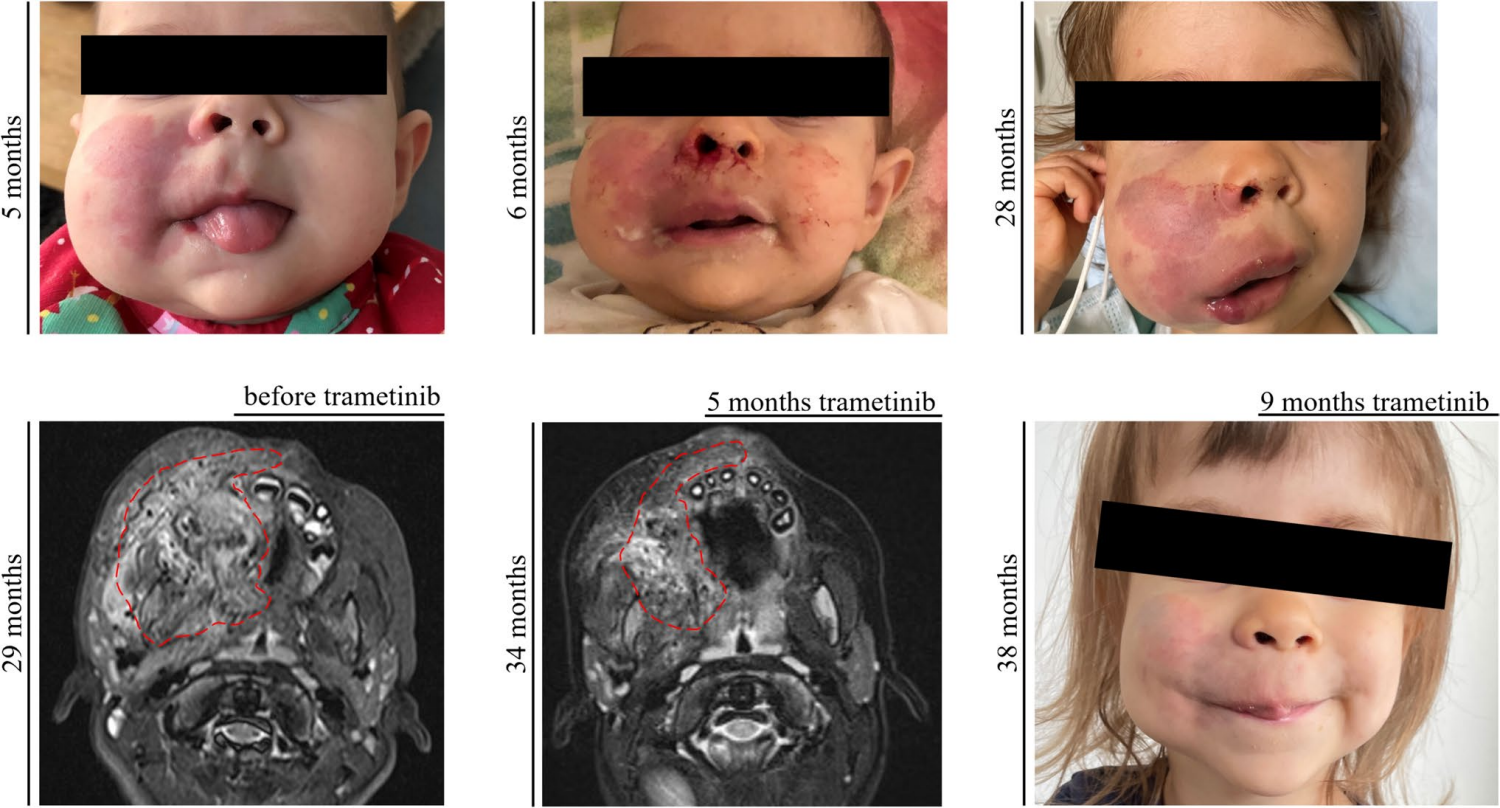
Response to targeted therapy in P1. **a** Patient P1 at 5 months of age.** b** Patient P1 at 6 months of age (after a bleeding episode).** c** Patient P1 at 28 months of age, showing a significant and progressive enlargement of the right cheek due to the AVM. **d** MRI (transversal T2 STIR) of the Patient P1 before the start of trametinib treatment; red dashed line indicates the extent of the AVM. **e** MRI (transversal T2 TSE Dixon) of the Patient P1 after 5 months of treatment with trametinib; red dashed line indicates the now smaller extent of the AVM.** f** Patient P1 at 38 months of age, reduced volume of the right cheek and fading of the capillary malformation can be noted

As described above, P1 had a refractory disease with recurrent life-threatening bleeding episodes. Due to the aggressive course of the disease, treatment with thalidomide [20] was started but was only transiently effective before symptoms deteriorated again (Fig. 5A–C). The MRI showed a large AVM of the right side of the face that was progressive over time (Fig. 5D and Online Resource 8). Because of increasing disease severity, off-label treatment with trametinib (0.25 mg per day (1/2 capsules), 0.023 mg/kg/d) was started at the age of 2 years and 6 months. Dosage was increased to 0.5 mg per day (1 capsule), 0.045 mg/kg body weight after one month. Trametinib led to a significant clinical response with a decrease in frequency and severity of bleeding episodes, a regression of the AVM as observed in the MRI (Fig. 5E), and a shrinking of the affected cheek (Fig. 5F). Due to improved disease control, the patient was able to attend preschool for the first time in her life. The patient tolerated trametinib treatment without significant adverse events and remained on this therapy for 9 months. At 2 years and 10 months of age, while on trametinib, the patient developed spontaneous rhinoliquorrhea and was diagnosed with a frontoethmoidal encephalocele, which, retrospectively, was already present in the first MRI at 6 months of age. She then underwent three neurosurgical operations; however, the cerebrospinal fluid (CSF) leak persisted and at the age of three years a ventriculoperitoneal (VP) shunt was implanted. After the placement of the VP shunt, the rhinoliquorrhea stopped. The VP shunt was replaced after 3 months due to a defect of the parietal skin overlying the shunt line. One month later, the patient developed a pneumococcal meningitis with cerebral edema and herniation leading to death at the age of 3 years and 3 months. We hypothesize that the frontoethmoidal encephalocele with difficult dural closure was a predisposing factor for this lethal infection but cannot rule out a contribution of trametinib treatment to this event. However, due to the absence of other adverse events (such as neutropenia or skin toxicity), and the safety profile of trametinib that does not include immunosuppressive effects, we consider this tragic fatal event after 9 months of trametinib treatment as unrelated to this medication.

## Discussion

In this report, we describe three novel somatic activating *RIT1* delins variants in patients with peripheral fast-flow malformations. The exact prevalence of *RIT1* variants as the cause for AVM cannot be determined precisely. However, with a total of three cases in the cohorts of vascular anomaly patients studied by this consortium, they are evidently much less common than variants of *MAP2K1* or *KRAS*. Considering the total number of AVMs sequenced and of AVMs harbouring a *RIT1* mutation, we roughly estimate the prevalence of *RIT1* mutations in AVMs at approximately 1 out of 30. Nevertheless, we strongly recommend that *RIT1* should be included in panels for genetic testing of patients with vascular malformations.

All three somatic mutations are located close to the switch 2 domain of *RIT1*, a domain that also harbors *RIT1* germline mutations commonly associated with Noonan syndrome. Interestingly, similar delins at the switch 2 domain of the RAS GTPases *KRAS* and *HRAS* have been described in vascular anomalies previously [21]. *RIT1* delins led to a strong ERK hyperphosphorylation that was much more pronounced than in Noonan-associated RIT1 missense changes. Considering the presumed role of RIT1 in the RAS-MAPK signaling pathway, treatment with a SHP2 inhibitor—as expected—did not influence ERK phosphorylation in cells transfected with the delins. In contrast, MEK inhibition completely rescued ERK hyperphosphorylation. These data suggest that *RIT1* delins act through overactivation of the canonical RAS-MAPK pathway.

Additionally, we observed overactivation of AKT in our biochemical assay, hinting at a known but so far underappreciated interconnection and crosstalk between the RAS and the AKT/mTOR signaling pathways in vascular anomalies. AKT activation has also been observed in patients with loss-of-function mutations in RASA1 (an inhibitor of the RAS pathway), where a consistent endothelial overactivation of mTORC1 could be found [22]. This is in line with data from brain AVMs due to KRAS mutations [23] as well as in other diseases, in which KRAS mutations activate the mTOR signaling pathway [24]. While there is little published data on the use of sirolimus in patients with AVM, it is currently not considered a viable option by experts in the field of vascular anomalies. A larger study on sirolimus for different kinds of vascular anomalies found only a very low rate of responders [25]. We hypothesize that *RIT1* delins close to the switch 2 region led to a distinct biochemical profile compared to missense mutations of Noonan syndrome, namely a stronger hyperphosphorylation of ERK, and—to a lesser extent—a phosphorylation of AKT at position T308. Exploring these functional differences of different mutations and the interaction of the RAS and the AKT/mTOR pathways might provide relevant insights into disease pathophysiology in the future.

To further assess the impact of the novel *RIT1* variants on vascular development, we used an approach that mimics the endothelial mosaicism that occurs in patients. In our previous work, we assessed various *TEK* mutations by endothelial-specific mosaic expression in zebrafish embryos and observed the development of venous malformations [18]. In the current work, we observed that *RIT1* mutations induced AVM-like lesions in zebrafish embryos, further confirming that overactivation of RAS-MAPK signaling caused by *RIT1* delins has a deleterious effect on vascular development. We refer to these lesions as AVM-like, as we observe a fast-flow shunt and a fusion of artery and vein but are still studying the biology of these shunts in greater detail in order to better understand the degree of similarity with AVMs in patients. Additionally, we show that MEK inhibition not only normalized ERK phosphorylation in vitro but also restored normal vascular development and decreased AVM-like lesion size in vivo during early and late treatment, respectively. These data further validate RAS-MAPK hyperactivation as the major driving mechanism for AVM formation and maintenance in the presence of the novel *RIT1* delins variants.

Trametinib is a mitogen-activated protein kinase (MAPK) kinase (MEK) inhibitor and as such an inhibitor of the RAS signaling pathway. By specifically binding to MEK1 and MEK2, trametinib inhibits the growth factor-mediated cell signaling and cellular proliferation in various cancers [26]. Due to the identification of the underlying mutations of the RAS signaling pathway in patients with AVMs [27–29] and certain complex lymphatic anomalies [30–32], trametinib has been used in patients with these diseases as an experimental drug with some success. Larger case series or even clinical trials studying the effect of trametinib on vascular anomalies driven by RAS activation have not been studied so far. To our knowledge, two clinical trials are currently registered at clinicaltrials.gov, which will study the effect of trametinib on AVMs prospectively (NCT04258046, NCT06098872), with additional trials using alternative MEK inhibitors such as cobimetinib (NCT05125471).

Our in vitro and in vivo findings and the available literature encouraged us to the off-label use of trametinib in our severely affected patient P1, who indeed responded very well with a significant reduction of AVM size and associated complaints, such as bleeding episodes. Unfortunately, the patient developed fatal meningitis, most likely due to an incidental encephalocele. While we consider this event as not related to trametinib treatment, it further highlights the need for controlled studies in the field of vascular anomalies, to assess treatment efficacy and tolerability and to advance care for patients with these diseases into an era of evidence-based personalized medicine.

In summary, our work introduces *RIT1* as a novel gene implicated in the pathogenesis of AVM. Functional testing in vitro and in vivo demonstrated the capacity of the novel *RIT1* variants to hyperactivate the RAS-MAPK pathway and induce the development of AVM. MEK inhibition led to biochemical normalization, prevention of AVM formation, as well as a decreasing AVM size. We also present the first promising data on the use of trametinib in a patient with a somatic *RIT1* mutation, encouraging further investigation of MEK inhibition in patients with AVM in future clinical trials. However, our n = 1 approach in this study precludes definitive conclusions regarding treatment efficacy and safety. Larger-scale studies are needed to validate our findings, to delineate a more precise estimation of the prevalence of *RIT1* mutations, to characterize their functional consequences on the RAS pathway and neighboring signaling pathways, and to assess the broader applicability of MEK inhibition in patients with RIT1-mutated vascular malformations.

## Materials and methods

### Patients/study approval

All subjects, and/or their legal guardians, gave written informed consent to genetic investigations, which were carried out with approval by the institutional review boards of the University Hospital Regensburg, Germany (17-854-101), University Hospital of Bern, Switzerland (2017-01960), and Boston Children’s Hospital, Boston, MA, USA (IRB-P00025772).

### Genetic testing

*RIT1* was tested in a total of 691 samples by the partners’ laboratories (235 in Magdeburg, 114 in Bern, 342 in Boston), including all types of vascular anomalies. Out of these samples, 118 were submitted for sequencing with the diagnosis of “AVM” (58 to Magdeburg, including by non-specialized centers, 35 to Bern, 25 to Boston). Brain AVMs were not included in the submitted samples. From these samples, one sample at each center harbored a *RIT1* mutation. Since sequencing results at Magdeburg also revealed mutations (e.g. in *TEK* or *GNAQ*) that according to current knowledge do not occur in AVMs, we conclude that phenotyping by non-specialized centers was in part incorrect and estimate that roughly 30–40 true AVM samples were sequenced in Marburg. This would be in line with a recently published cohort from Germany that included 29 patients with mutations in the RAS pathway [33]. We thus estimate the prevalence of *RIT1* mutations in AVMs at roughly 1 in 30 patients.

A tissue biopsy of the AVM of P1 was submitted to the Institute of Human Genetics, University Hospital Magdeburg, and the genomic DNA was extracted. Assuming a mosaic mutation as the cause of the disorder, ultradeep sequencing and enrichment using an Agilent SureSelect XT HS2 Custom Enrichment Panel with molecular barcoding (UMIs, 3 bp duplex) (Agilent Technologies) were performed. The library was sequenced on a NextSeq550 instrument (Illumina), 2 × 150 bp paired-end reads. The target regions had a mean coverage of > 3000× after demultiplexing. The varvis 1.20.0 analysis software (Limbus Medical Technologies GmbH) was used for analysis.

P2 has been included in the Bernese Congenital Vascular Malformation Registry, a prospective cohort of congenital extracranial/extraspinal vascular malformations that have been enrolling consecutive patients since 2008 [34]. As of October 2020, genetic testing is performed on tissue available from diagnostic biopsies of vascular malformations, using the TruSight Oncology 500 (TSO500; Illumina) Next Generation Sequencing (NGS) gene panel.

Resected tissue from P3 underwent targeted DNA NGS testing via the OncoPanel assay at the Center for Advanced Molecular Diagnostics (CAMD) at Brigham and Women’s Hospital [35]. DNA was isolated using standard extraction methods (QIAGEN) and quantified with PicoGreen-based double-stranded DNA detection (Thermo Fisher Scientific). Indexed sequencing libraries were prepared from 50-ng sonically sheared DNA samples using Illumina TruSeq LT reagents (Illumina). Extracted DNA underwent targeted NGS using the KAPA HTP Library Preparation Kit (Roche), a custom RNA bait set (Agilent SureSelect) and sequenced with the Illumina HiSeq 2500 system.

### Cell culture and western blot

Three million HEK293T cells were seeded in 10 cm cell culture plates supplemented with DMEM containing 10% fetal bovine serum (FBS) 12 h prior to transfection. At around 70% confluency levels cells were transfected using TurboFect transfection reagent (Thermo Fisher #R0532), with Flag-tagged *RIT1* variants in pCDNA constructs or empty vector (EV) as the negative control. The medium was refreshed at the 24 h’ time point with MEK inhibitor (PD0325901, Selleckchem # S1036) and SHP2 inhibitor (SHP099, Selleckchem # S6388) being added to the transfected cells at 1 μM and 5 μM concentrations, respectively. At 48 h post-transfection, cells were washed in ice-cold phosphate-buffered saline (PBS) and lysed in ice-cold lysis buffer, containing 50 mM Tris/HCl pH 7.5, 5 mM MgCl2, 100 mM NaCl, 1% Igepal CA-630, 10% glycerol, 20 mM ß-glycerolphosphate, 1 mM Na-orthovanadate, EDTA-free inhibitor cocktail 1 tablet/50 ml. After the addition of Laemmli sample buffer, the samples were subjected to SDS-PAGE (12.5% polyacrylamide). Blots were detected by immunoblotting using a mouse anti-γ-Tubulin antibody (Sigma #T5326), a mouse anti-FLAG antibody (Sigma #F3165), a rabbit anti-ERK antibody (Cell signaling technology #9102), and a rabbit anti-p-ERK antibody (Cell signaling technology #4370), a rabbit anti-AKT (Cell signaling technology #9272), a rabbit anti-p-AKT-Thr308 (Cell signaling technol-ogy #2965), a rabbit anti-p-AKT-Ser473 (Cell signaling technology #4060). The immunoblots were detected using an Odyssey Fc Imaging System (LI-CORE Biosciences) and analyzed by Image Studio Lite Ver 5.2.

### Zebrafish husbandry

Maintenance and breeding of zebrafish (*Danio rerio*) were performed in the fish facility of the Developmental Biology, Institute for Biology I, University of Freiburg under standard conditions. Only embryos up to 5 days post-fertilization were used. All experiments were carried out in accordance with German laws for animal care and the Regierungspräsidium Freiburg.

### Plasmid preparation

Plasmids were designed using ApE—A plasmid editor version 3.0.8. *Homo sapiens RIT1* sequence was obtained from the online database Ensembl (Transcript ID: ENST00000368323.8), minimally codon optimized for *Danio rerio* and ordered as a plasmid including Tol2 sites, a UAS promoter, RIT1^P2^, and P2A-GFP from Twist Bioscience (South San Francisco, CA, USA). Plasmids were purified using Wizard Plus SC Minipreps DNA Purification Systems (Promega, Walldorf, Germany, A1330) according to the manufacturer’s instructions.

### Mutagenesis

*RIT1*^*wt*^, empty vector (EV), as well as all other *RIT1* mutations analyzed in this study were derived from the UAS:RIT1^P2^-P2A-GFP construct using Q5 Site-Directed Mutagenesis (New England Biolabs, E0554S). Corresponding mutagenesis primers were designed using NEBaseChanger version 1.3.3. All plasmids were sequenced by Eurofins genomics to confirm the expected sequence.

### Tol2 transposase mRNA synthesis

8 µg of the plasmid that contains the transposase gene under control of the SP6 promoter were linearized using 4 µl of NotI-HF enzyme (New England Biolabs) for 1 h at 37 °C. The digested sample was purified using the QIAquick PCR Purification Kit according to the manufacturer’s protocol. Capped Tol2 transposase mRNA was synthesized from purified DNA using the mMESSAGE mMACHINE™ SP6 Transcription Kit (ThermoFisher Scientific) according to the manufacturer’s protocol. The resulting mRNA was separated into 5 µl aliquots and stored at − 20 °C to prevent freeze–thaw cycles.

### Plasmid injection

The construct was then injected into *Tg*(*fli1a:Gal4FF*^*ubs3*^*; UAS:RFP)* embryos at the one-cell stage together with Tol2 transposase mRNA [36], both at a concentration of 30 ng/µl. For better readability, *Tg*(*fli1a:Gal4FF*^*ubs3*^*; UAS:RFP)* embryos injected with a gene of interest (e.g. UAS:RIT1^P1^-P2A-GFP) are abbreviated as *fli1a:RIT1*^*P1*^_*G0mosaic*_ instead of *Tg*(*fli1a:Gal4FF*^*ubs3*^*; UAS:RFP)* and *Tg(UAS:RIT1*^*P1*^*-P2A-GFP)*_*G0mosaic*_.

### Microscopy

Imaging plates for confocal microscopy were prepared in 35 mm glass bottom dish with 1.5% agarose in egg water, 1-phenyl 2-thiourea (PTU) and tricaine mix using previously designed and 3D printed molds (Online resource 9). Embryos anesthetized with 0.168 mg/ml tricaine in egg water at room temperature and gently positioned laterally inside the trenches. Confocal microscopy images were acquired as z-stacks with ZEISS Celldiscoverer 7 with LSM 900. Images were obtained with the 488 nm and 561 nm lasers, with a slice interval of 2–4 μm with a 20X (NA 0.7) objective or as brightfield images with 5x (NA 0.35) objective unless otherwise specified. For brightfield timelapse experiments, the interval was set to 1 s.

Fluorescence microscopy images for pre and post late treatment was acquired with ZEISS Axio Examiner D.1 fixed stage fluorescence microscope. During acquisition embryos were placed in 3 ml of E3 medium with tricaine (0.168 mg/ml) at room temperature and imaged with 10X (NA 0.15) objective. During imaging both, RFP and GFP channels acquired and only RFP channel is exported for representative images.

Lightsheet microscopy was performed with ZEISS Lightsheet 7 using water immersion W Plan-Apochromat 10x (NA 0.5) M27 75 mm objective. Images were obtained with the 488 nm and 561 nm lasers using single illumination, pivot scan on, with a slice interval of 2 μm. Timelapse interval is set to 1 s.

The color cyan was assigned for GFP channel. Colored versions of the images are included in the supplementary information. Images and videos were exported as.tif and.AVI (uncompressed) files, respectively using Fiji software.

### Pharmacological treatments

For pharmacological treatments, injected zebrafish embryos were randomized into control and treatment groups. From the 10-somite stage or from 48 hpf on, embryos of the treatment group were transferred in E3 Medium (5 mM NaCl, 0.17 mM KCl, 0.33 mM CaCl_2_, 0.33 mM Mg_2_SO_4_) containing 0.2 mM 1-phenyl 2-thiourea (PTU; Sigma, Taufkirchen, Germany, P7629) and MEK1/2 inhibitor Trametinib (MedChemExpress, GSK1120212; 10 mg) using 100× stock solutions dissolved in dimethyl sulfoxide (DMSO; Sigma, D2650). The treatment dose of trametinib was chosen at 100 nM, according to our previous publication [18], and by repeating of the toxicity assay in zebrafish embryos. Embryos of the control group were raised in E3 medium with 0.2 mM PTU and DMSO (equal amount to the treatment group). The response of the AVM-like lesion size to trametinib was calculated as follows: each embryo with an AVM-like lesion was imaged at the Axio Examiner, and the area of the lesion was divided by the area of the entire embryo to give a relative area of the malformation at 2 dpf. This was done to control for different embryo sizes and different embryo growth rates. This measurement was then repeated at 4 dpf (after treatment) and the relative area of the AVM-like lesion at 4 dpf was divided by the relative area 2 dpf, followed by multiplication with 100 to give a result in percent. A result greater than 100% showed an AVM-like lesion growing in size in relation to the embryo, a result less than 100% showed a regressing lesion.

### Statistical analysis

The statistical analysis was performed using two-tailed Fisher’s exact test of significance for malformation rate analysis and early pharmacological treatments and two-tailed unpaired t-test for late pharmacological treatment experiments and one-way ANOVA for in vitro experiments in GraphPad Prism version 10.2.2. The legends of the figures include information on sample sizes and significance. P value of < 0.05 was regarded as significant. Analyzed data for zebrafish malformation rates and early pharmacological treatments was obtained from at least three independent experiments for each variant. The number of examined embryos is indicated in the figure legends for each variant. Data are presented as mean ± SD for all experiments. P value *< 0.05, **< 0.01; ***< 0.001; **** < 0.0001. The structure of the RIT1 protein is predicted using AlphaFold database [37, 38].

## Supplementary Information

Below is the link to the electronic supplementary material.

Supplementary file1 (PDF 809 KB)

Supplementary file2 (AVI 52115 KB)

Supplementary file3 (AVI 41981 KB)

Supplementary file4 (AVI 108069 KB)

Supplementary file5 (AVI 1057 KB)

Supplementary file6 (STL 132 KB)

Supplementary file7 (PDF 2182 KB)

Supplementary file8 (XLSX 227 KB)

## Data Availability

The data that support the findings of this study are available from the corresponding author upon reasonable request.

## Abbreviations

AVM: Arteriovenous malformation
CSF: Cerebrospinal fluid
dpf: Days post fertilization
hpf: Hours post fertilization
PTU: 1-Phenyl 2-thiourea
ISSVA: International Society for the Study of Vascular Anomalies
MRI: Magnetic resonance imaging
NGS: Next generation sequencing
VP: Ventriculoperitoneal
