# Supplementary material for: Somatic RIT1 delins in arteriovenous malformations hyperactivate RAS-MAPK signaling amenable to MEK inhibition
Source: Angiogenesis. 2024 Jul 5;27(4):739–52. doi: 10.1007/s10456-024-09934-8 (PMC11564399; doi:10.1007/s10456-024-09934-8)
Supplement: Supplementary file 1 — Supplementary file1 (PDF 809 KB) [file 10456_2024_9934_MOESM1_ESM.pdf]

Supplementary Information

to

Somatic RIT1 delins in arteriovenous malformations hyperactivate RAS-MAPK signaling amenable to MEK inhibition

Journal name: Angiogenesis

Authors:

Friedrich G. Kapp[1 #*], Farhad Bazgir[2 *], Nagi Mahammadzade[1 *], Mehrnaz Mehrabipour[2], Erik Vassella[3], Yvonne Döring[4,5,6], Annegret Holm[1,7], Axel Karow[8], Caroline Seebauer[9], Natascha Platz Batista da Silva[10], Walter A. Wohlgemuth[11], Aviv Oppenheimer[1], Pia Kröning[12], Charlotte M. Niemeyer[1], Denny Schanze[13], Martin Zenker[13], Whitney Eng[14], Mohammad R. Ahmadian[2], Iris Baumgartner[4,5], Jochen Rössler[1,15,16 #]

[1]Division of Pediatric Hematology and Oncology, Department of Pediatrics and Adolescent Medicine, Medical Center-University of Freiburg, Faculty of Medicine, University of Freiburg, 79106 Freiburg, Germany, VASCERN VASCA European Reference Centre

[2]Institute of Biochemistry and Molecular Biology II, Medical Faculty and University Hospital, Heinrich-Heine University, Düsseldorf, Germany

[3]Institute of Pathology and Tissue Medicine, University of Bern, Bern, Switzerland

[4]Division of Angiology, Swiss Cardiovascular Center, Inselspital, Bern University Hospital, Bern, Switzerland

[5]Department for BioMedical Research (DBMR), University of Bern, Switzerland

[6]Institute for Cardiovascular Prevention (IPEK), Ludwig-Maximilians University Munich, Pettenkoferstr 9, 80336, Munich, Germany

[7]*Vascular Biology Program, Department of Surgery, Boston Children's Hospital, Harvard Medical School, Boston, MA, United States.*

[8]*Department of Pediatrics and Adolescent Medicine, Friedrich-Alexander-Universität Erlangen-Nürnberg (FAU), D-91054 Erlangen, Germany*

[9]*Department of Otorhinolaryngology, Regensburg University Medical Center, Franz-Josef-Strauß-Allee 11, 93053 Regensburg, Germany*

[10]*Department of Radiology, Regensburg University Medical Center, Franz-Josef-Strauß-Allee 11, 93053 Regensburg, Germany*

[11]*University Clinic and Policlinic of Radiology at the Martin-Luther-Universität Halle-Wittenberg, Halle, Germany*

[12]*Department of General Pediatrics, Adolescent Medicine and Neonatology, Medical Center-University of Freiburg, Faculty of Medicine, University of Freiburg, 79106 Freiburg, Germany*

[13]*Institute of Human Genetics, University Hospital Magdeburg, 39120 Magdeburg, Germany*

[14]*Division of Hematology/Oncology, Boston Children's Hospital and Harvard Medical School, Boston, MA, USA*

[15]*Department of Vascular Medicine, National Reference Center of Rare Lymphatic and Vascular Diseases, UA11 INSERM – UM IDESP, Campus Santé, Montpellier Cedex 5, France*

[16]*Division of Paediatric Hematology and Oncology, Department of Paediatrics, Inselspital, Bern University Hospital, University of Bern, Switzerland*

*Contributed equally to this work

[#]*Corresponding authors Email: friedrich.kapp@uniklinik-freiburg.de;*

jochen.roessler@insel.ch

This file contains the following:

Additional Clinical and Laboratory Findings

- Supplementary Figures S1 (Online Resource 1)
- Supplementary Figures S2 (Online Resource 2)
- Supplementary Figure S3 (Online Resource 3)
- Supplementary Figure S4 (Online Resource 4)
- Supplementary Video S1 (Online Resource 5)
- Supplementary Video S2 (Online Resource 6)
- Supplementary Figure S5 (Online Resource 7)
- Supplementary Figure S6 (Online Resource 8)
- Supplementary File S1 (Online Resource 9)

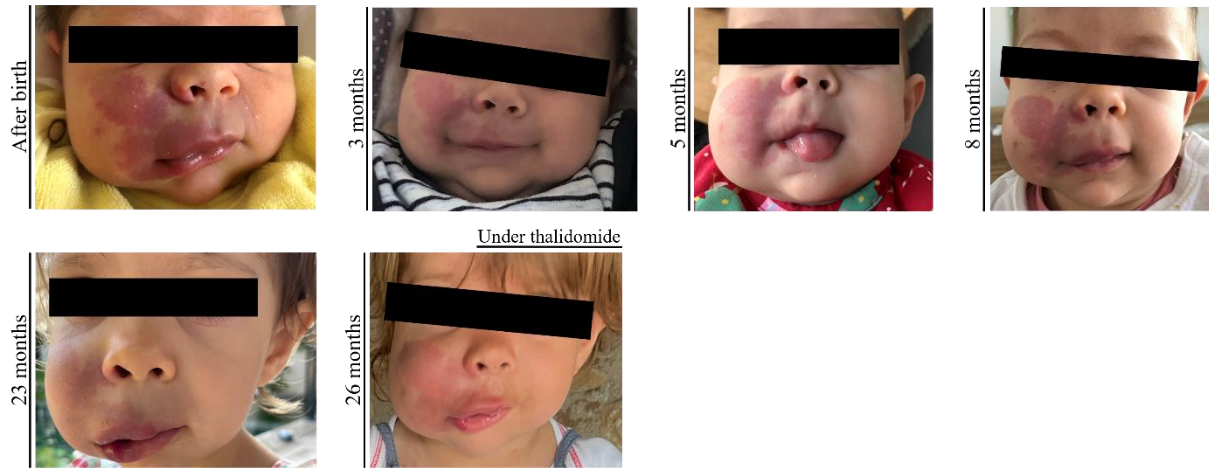

Supplemental Figure S1 (Online Resource 1). Patient P1 – Clinical evolution of disease.

Clinical photographs of Patient P1 show progressive growth of the lesion over time.

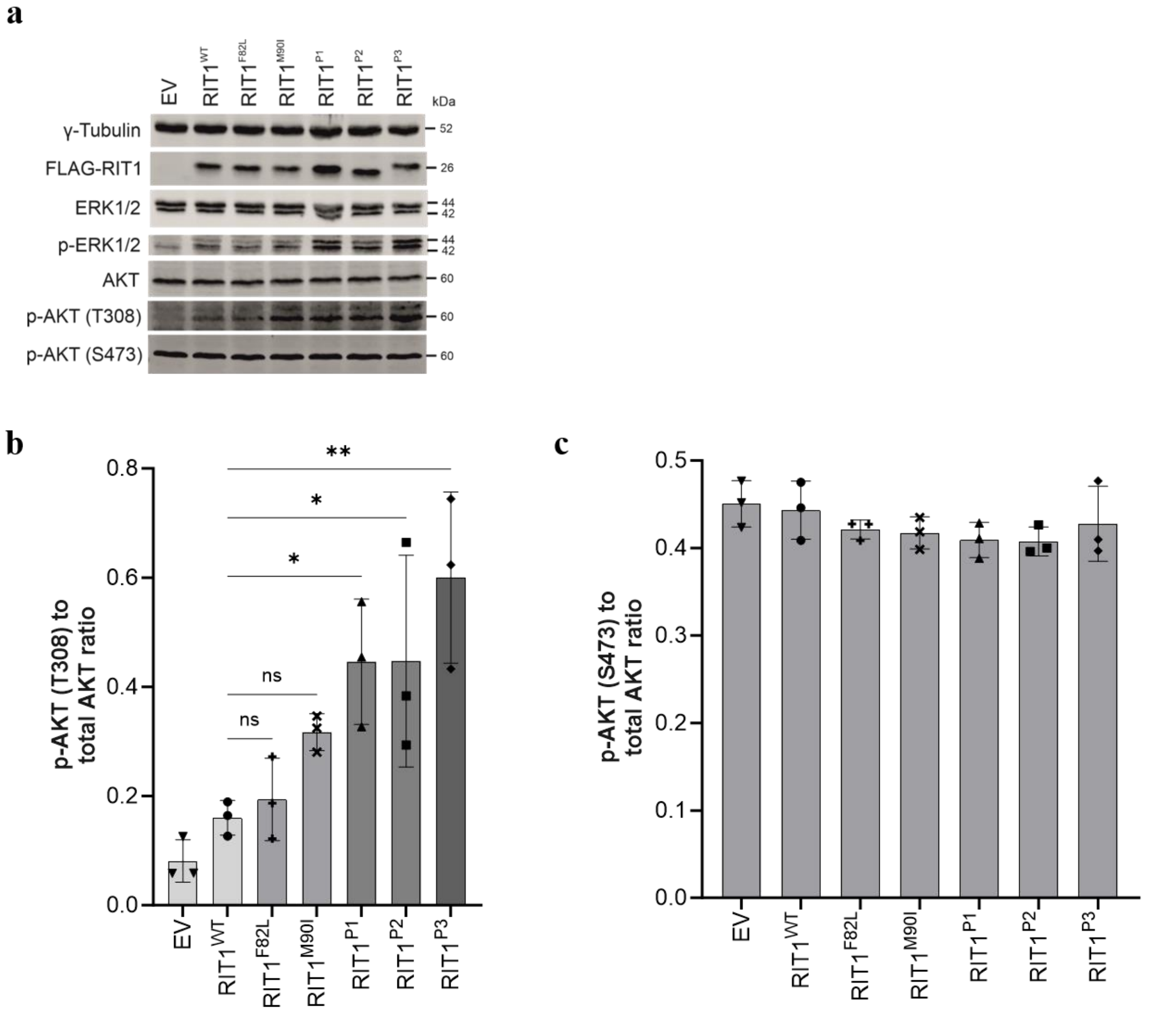

Supplemental Figure S2 (Online Resource 2). AKT phosphorylation after expression of *RIT1* variants *in vitro* in HEK293T cells.

a. Western blot after expression of *RIT1* variants to assess RAS-MAPK pathway and PI3K/AKT signaling pathway activation. Gamma tubulin served as loading control, FLAG-RIT1 confirms the expression of the construct, total ERK and AKT levels serve as a control to exclude the differential expression of ERK and AKT. p-ERK measures the level of phosphorylation of ERK as a marker of RAS pathway activation. p-AKT measures the level of phosphorylation of AKT as a marker of mTOR pathway activation.

b. Ratios of p-AKT (Thr308) as a substrate of PDK1 to total levels of AKT. Quantification of the AKT phosphorylation was measured in a total of three western blots (n=3). One-way ANOVA. P-value ∗<0.05, ∗∗<0.01. Data are presented as mean ± SD. EV = empty vector.

c. Ratios of p-AKT (Ser473) as the target of mTORC2 to total levels of AKT. Quantification of the AKT phosphorylation was measured in a total of three western blots (n=3). One-way ANOVA. The difference between groups is non-significant. Data are presented as mean ± SD. EV = empty vector.

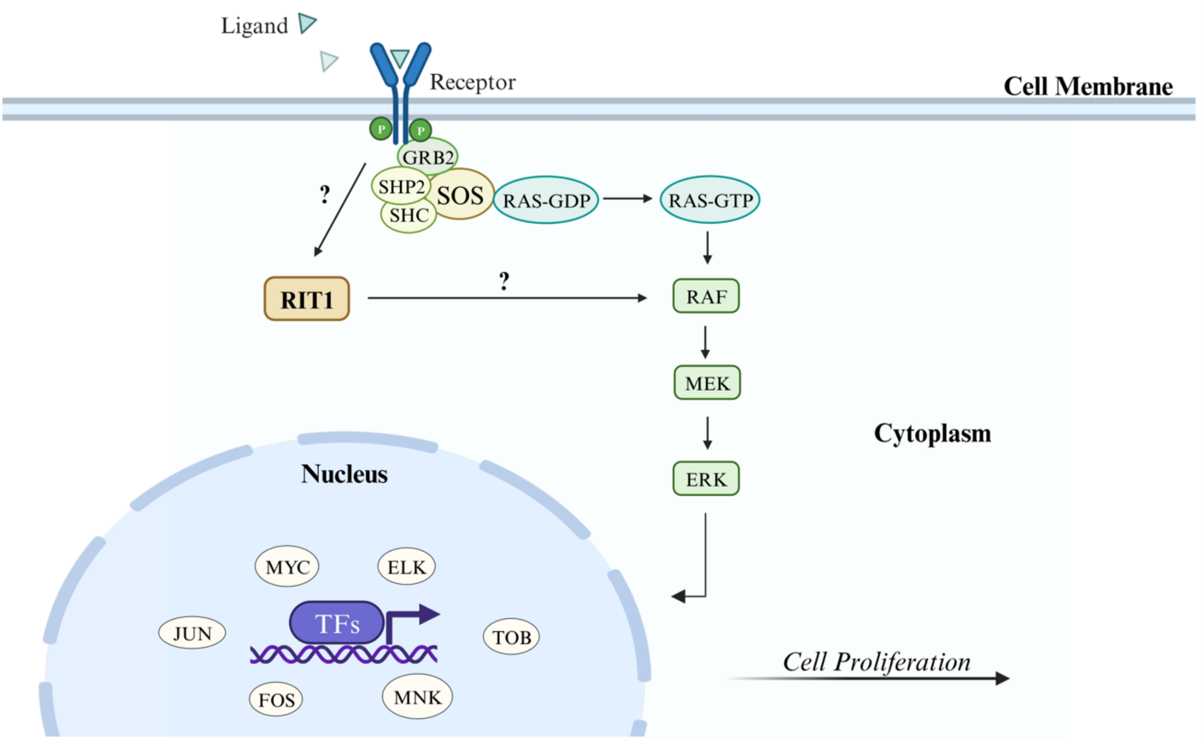

Supplemental Figure S3 (Online Resource 3). Schematic of the RAS signaling pathway.

SHP2 is upstream, MEK further downstream in the RAS-MAPK signaling pathway; created with BioRender.com.

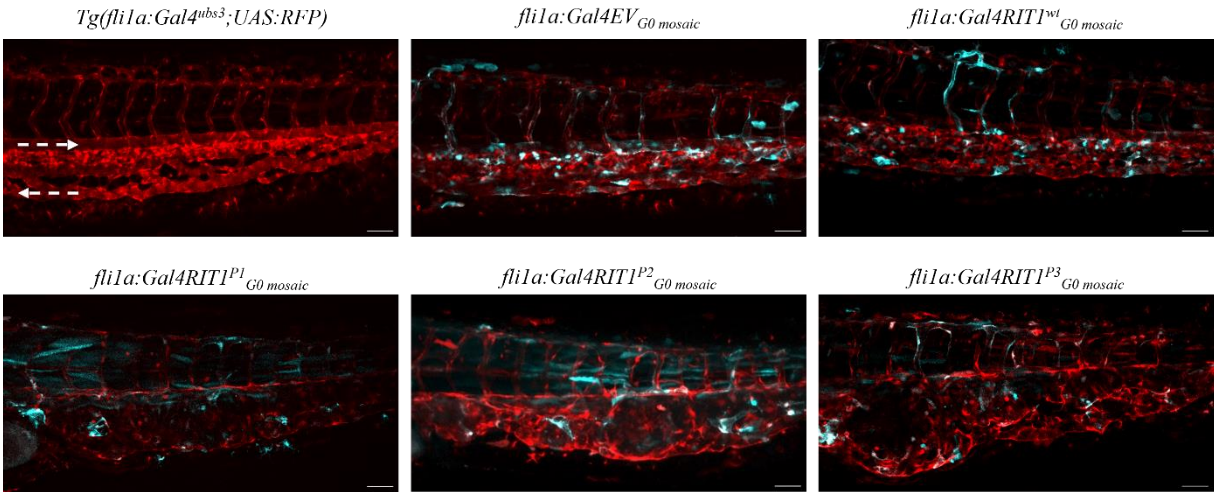

Supplemental Figure S4 (Online Resource 4). Figure 3b zebrafish AVM phenotype with assigned false colors.

Red color is assigned for RFP channel and cyan color is assigned for GFP channel. White arrows represent the direction of arterial (top) and venous blood flow (bottom) respectively. Scale bar 50 µm.

Supplemental Video S1 (Online Resource 5). Comparison of normal circulation and aberrant connection of aorta and caudal vein with fusion and dilation of vasculature in the tail distal to the AVM.

a. Notice that the blood in the dorsal aorta of the uninjected *Tg(fli1a:Gal4)* fish flows to the end of the tail and then returns in the caudal vein. Scale bar 50 µm.
b. Notice the aberrant flow in the dorsal aorta of the $RIT1^{P2}$ injected fish which moves into the caudal vein proximal in the tail, as well as a fusion of aorta and the upper part of the caudal vein plexus. Scale bar 50 µm.

Supplemental Video S2 (Online Resource 6). Positions of AV shunts in different zebrafish phenotypes.

a. Light-sheet time-lapse imaging of embryos with GFP tagged erythrocytes after *RIT1*^{P3} injection. *Tg(fli:Gal4, UAS:RFP)* fish crossed with *Tg(LCR:GFP)* line and *RIT1*^{P3} microinjections performed at the 1-cell stage. The shunt is only at the proximal end of the lesion but fusion of aorta and caudal vein plexus can also be observed distal to the shunt. Some erythrocytes sediment in the distal part of the lesion due to the proximal shunt and lack of blood flow in the distal part (and due to the upright position of the embryo during light-sheet microscopy). The size and pressure from the lesion are also prevents normal flow even in intersegmental vessels (ISVs). Scale bar 50 µm.
b. Example of an AV shunt (white arrow) after mosaic endothelial-specific expression of MAP2K1^{K57N} in *Tg(fli1a:Gal4; UAS:RFP)* fish line. Blood flow and vascular architecture distal to the shunt is completely normal, indicating that a proximal shunt is not sufficient to lead to abnormal vascular development in the tail. Angiography is performed with Dextran, Fluorescein, 500,000 MW (#D7136) at 2dpf. Scale bar 50 µm.

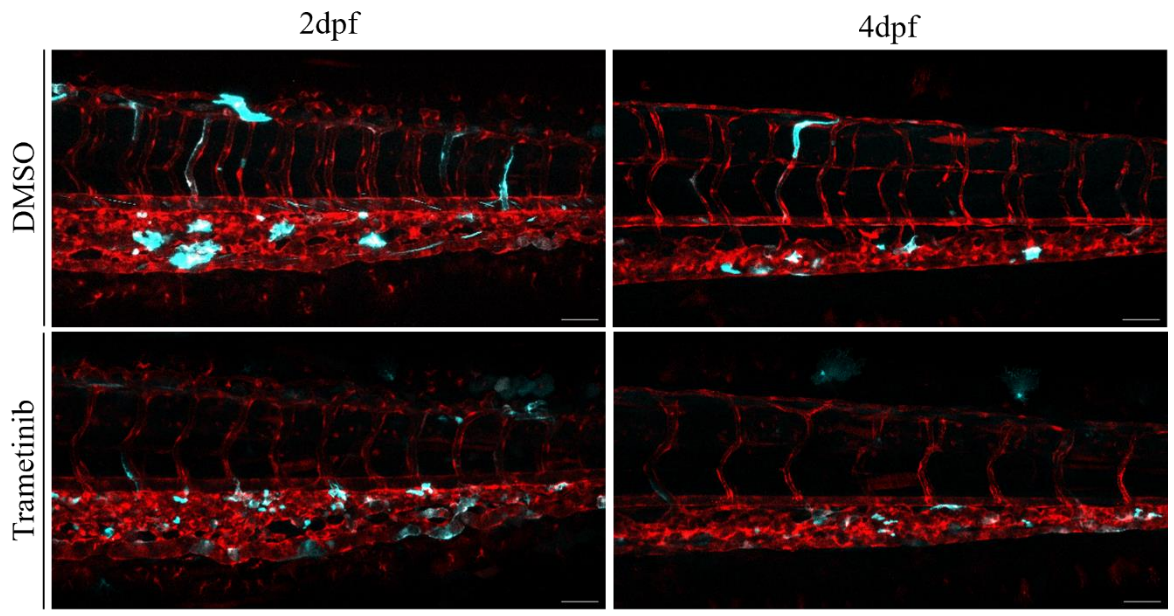

Supplemental Figure S5 (Online Resource 7). The effect of trametinib on empty vector injected embryos.

The effect of late trametinib treatment on empty vector injected embryos. Red color is assigned for RFP channel and cyan color is assigned for GFP channel. Scale bar 50 µm.

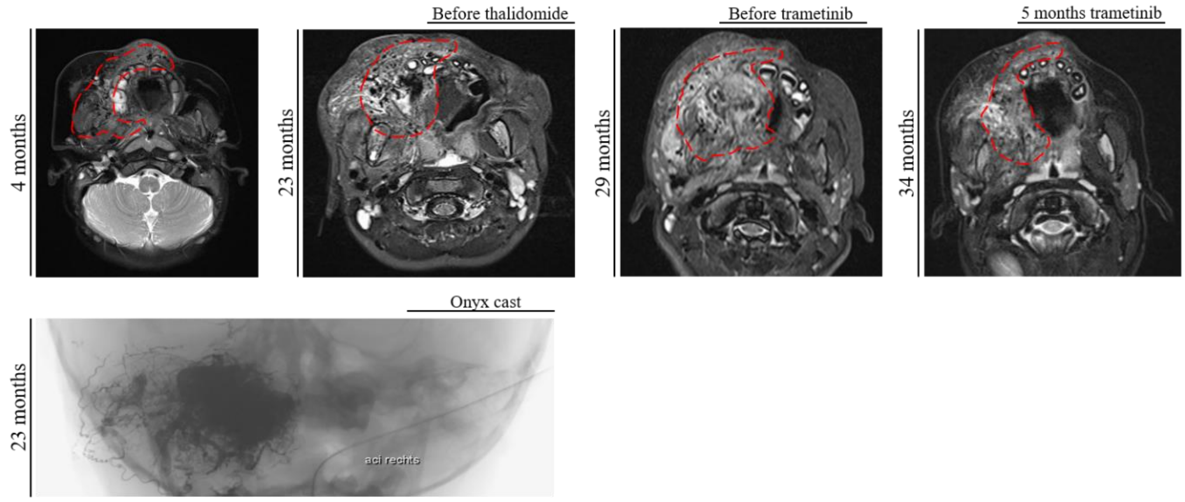

Supplemental Figure S6 (Online Resource 8). Patient P1 – Radiological evolution of disease.

(Upper panels) MRI images show the AVM's progression over time, non-response to thalidomide, and regression under trametinib treatment.

(Lower panel) Image of an angiography showing the extent of the Onyx cast on the right side of the face.

Supplemental File S1 (Online Resource 9). .STL file of the designed mould that is used to create imaging plates for Zeiss CD7 microscope.
