# Supplementary figures and images for: Somatic RIT1 delins in arteriovenous malformations hyperactivate RAS-MAPK signaling amenable to MEK inhibition
Source: Angiogenesis. 2024 Jul 5;27(4):739–52. doi: 10.1007/s10456-024-09934-8 (PMC11564399; doi:10.1007/s10456-024-09934-8)

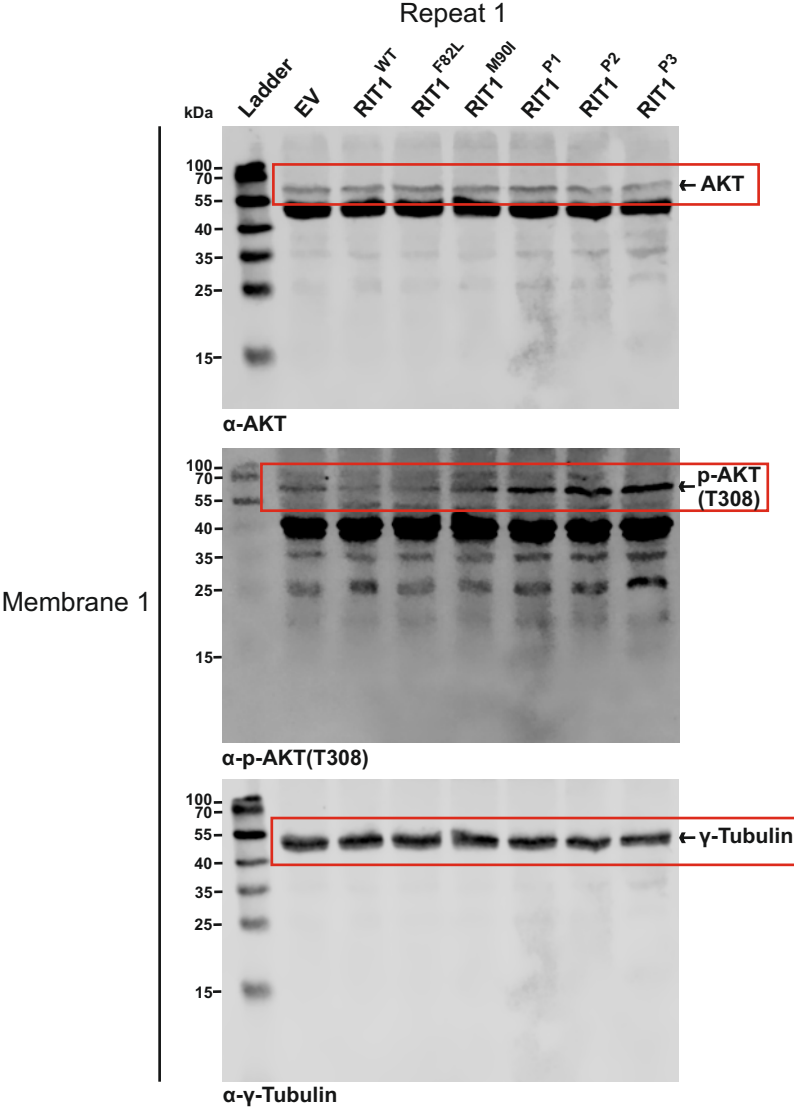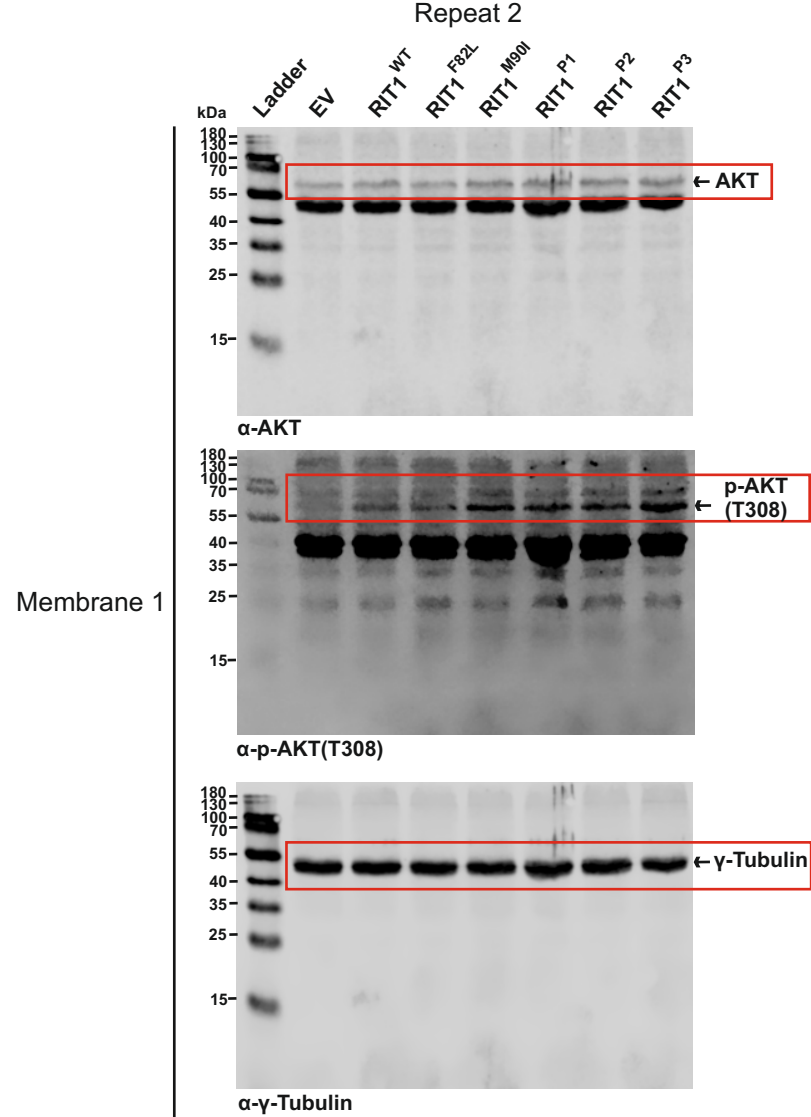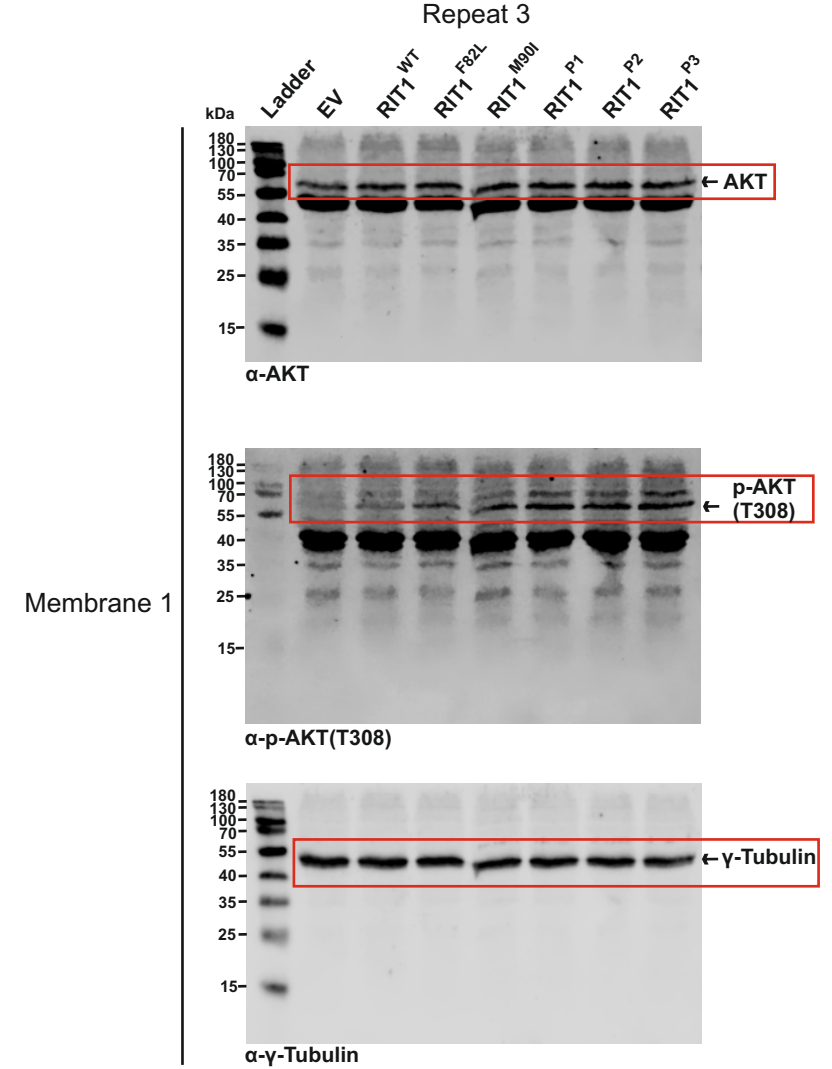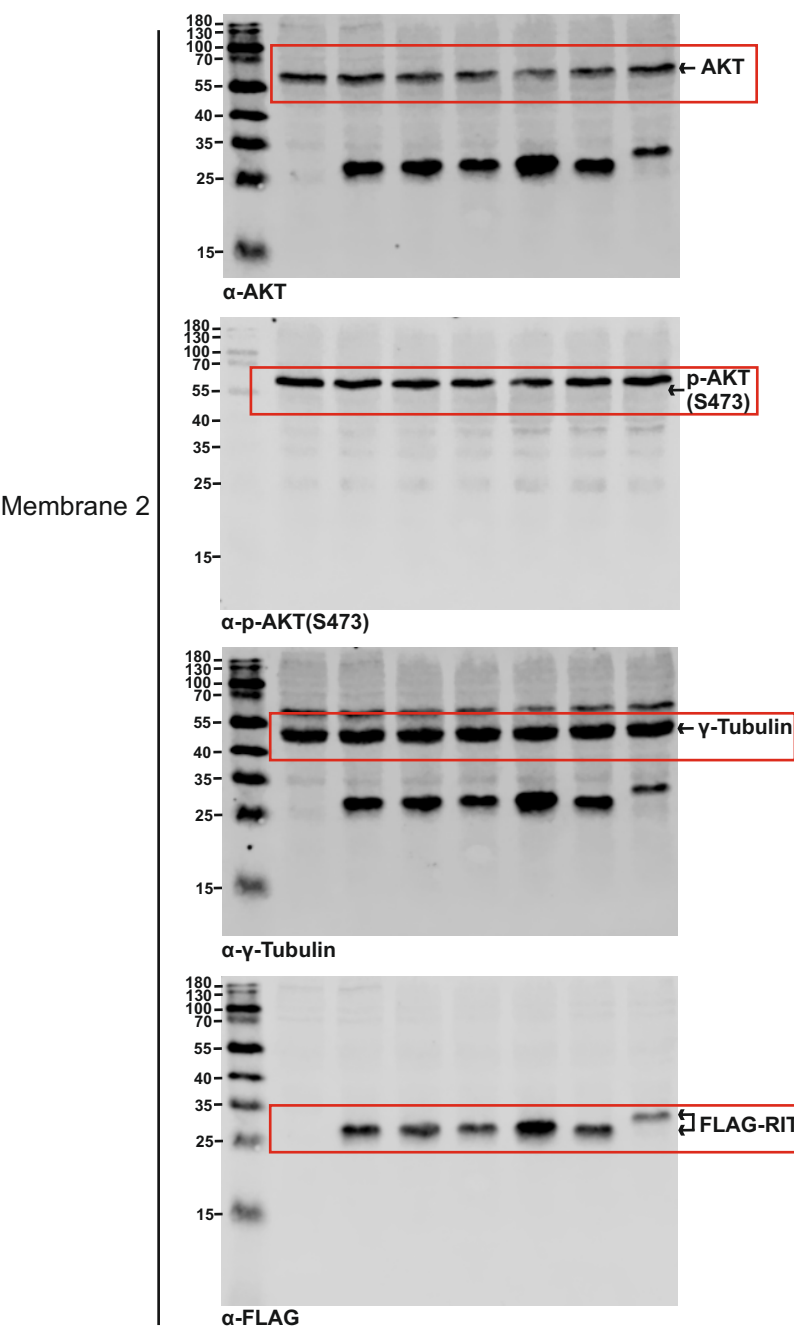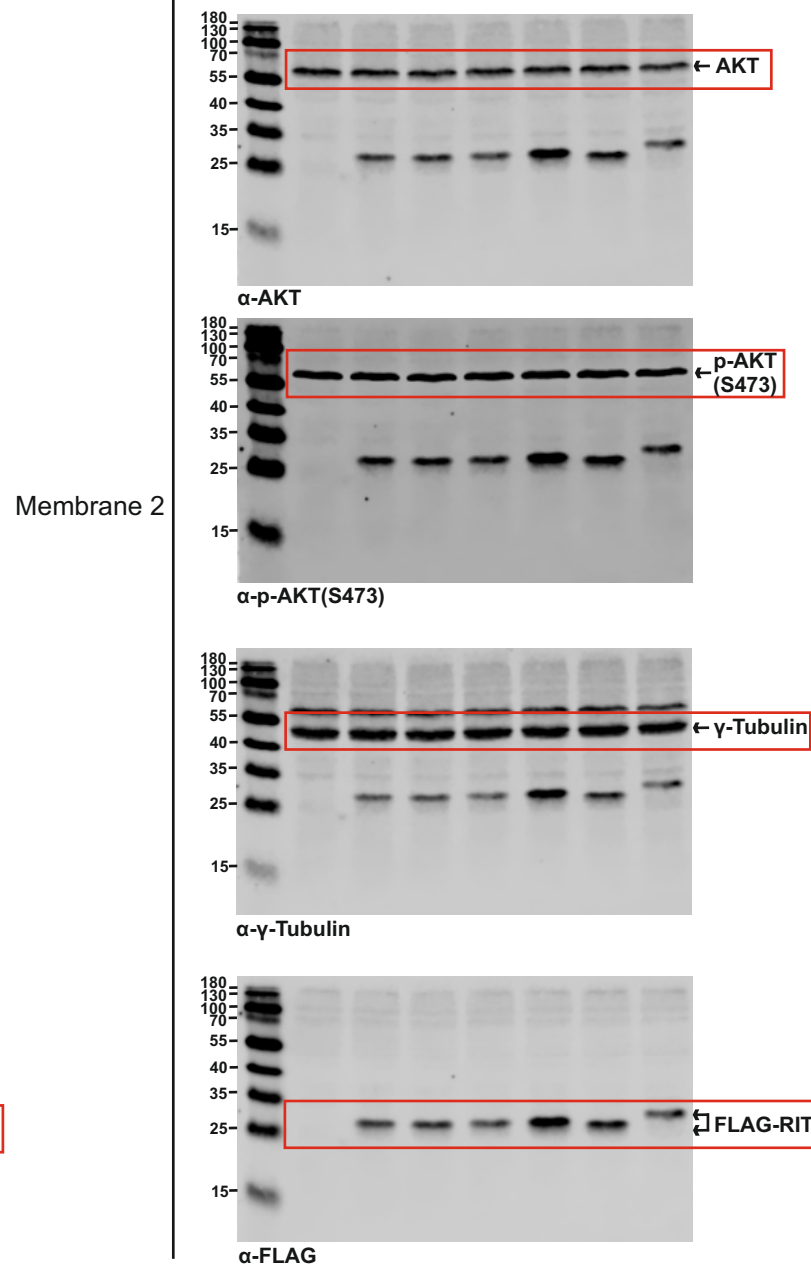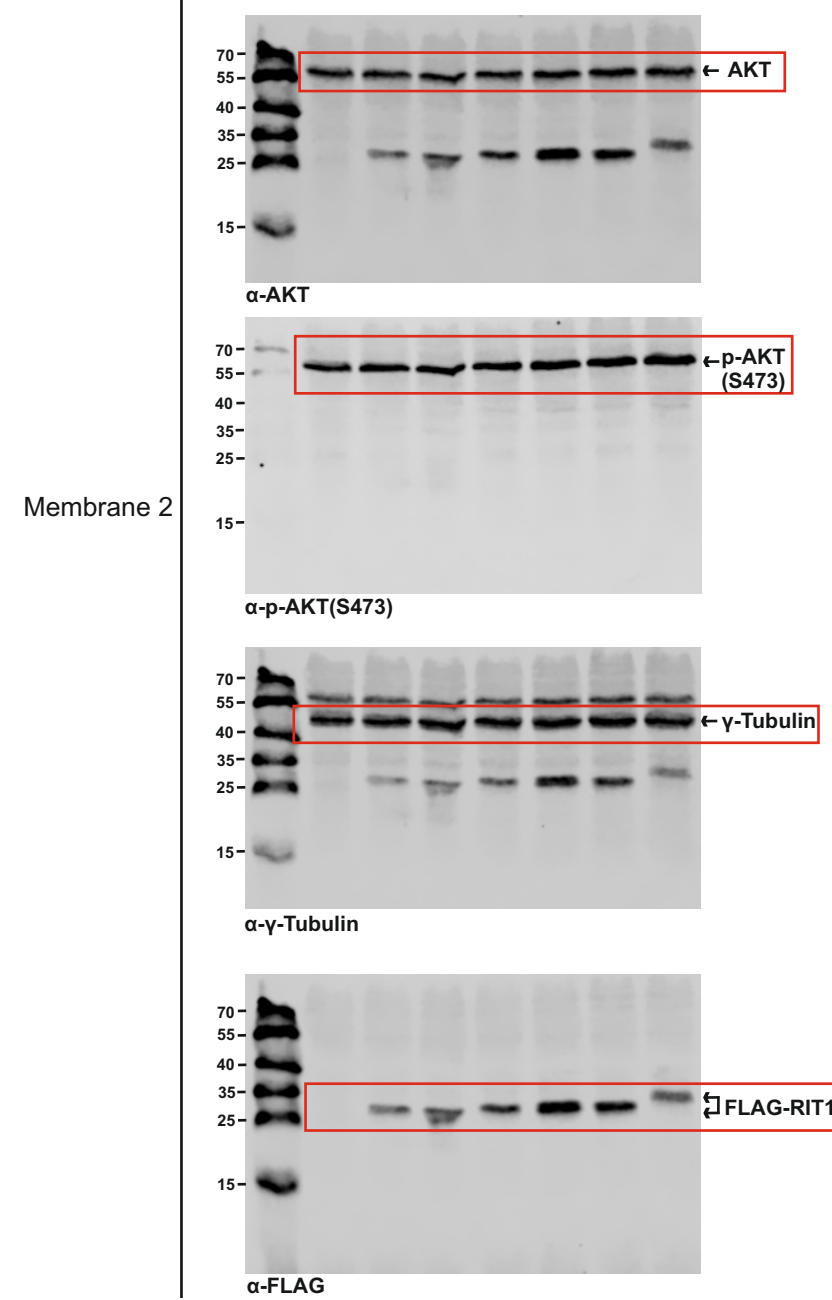

Supplement: Supplementary file 7 — Supplementary file7 (PDF 2182 KB) [file 10456_2024_9934_MOESM7_ESM.pdf]
